# Proposal to Improve the Image Quality of Short-Acquisition Time-Dedicated Breast Positron Emission Tomography Using the Pix2pix Generative Adversarial Network

**DOI:** 10.3390/diagnostics12123114

**Published:** 2022-12-09

**Authors:** Tomoyuki Fujioka, Yoko Satoh, Tomoki Imokawa, Mio Mori, Emi Yamaga, Kanae Takahashi, Kazunori Kubota, Hiroshi Onishi, Ukihide Tateishi

**Affiliations:** 1 Department of Diagnostic Radiology, Tokyo Medical and Dental University, Tokyo 113-8510, Japan; 2 Yamanashi PET Imaging Clinic, Chuo City 409-3821, Japan; 3 Department of Radiology, University of Yamanashi, Chuo City 409-3898, Japan; 4 Department of Radiology, Dokkyo Medical University Saitama Medical Center, Koshigaya 343-8555, Japan

**Keywords:** dedicated breast positron emission tomography, breast cancer, image synthesis, generative adversarial network, artificial intelligence

## Abstract

This study aimed to evaluate the ability of the pix2pix generative adversarial network (GAN) to improve the image quality of low-count dedicated breast positron emission tomography (dbPET). Pairs of full- and low-count dbPET images were collected from 49 breasts. An image synthesis model was constructed using pix2pix GAN for each acquisition time with training (3776 pairs from 16 breasts) and validation data (1652 pairs from 7 breasts). Test data included dbPET images synthesized by our model from 26 breasts with short acquisition times. Two breast radiologists visually compared the overall image quality of the original and synthesized images derived from the short-acquisition time data (scores of 1–5). Further quantitative evaluation was performed using a peak signal-to-noise ratio (PSNR) and structural similarity (SSIM). In the visual evaluation, both readers revealed an average score of >3 for all images. The quantitative evaluation revealed significantly higher SSIM (*p* < 0.01) and PSNR *(p* < 0.01) for 26 s synthetic images and higher PSNR for 52 s images (*p* < 0.01) than for the original images. Our model improved the quality of low-count time dbPET synthetic images, with a more significant effect on images with lower counts.

## 1. Introduction

Breast cancer (BC) is the most common cancer and the second leading cause of cancer-related deaths among women; moreover, its incidence is increasing globally [1]. [^18^F]fluorodeoxyglucose (^18^F-FDG) positron emission tomography (PET)/computed tomography (CT) is widely used for identifying distant metastases and secondary cancers, staging and monitoring treatment response [2,3,4]. In addition, some studies have reported using PET/CT for predicting the prognosis of patients with BC [5,6].

Recently, dedicated breast PET (dbPET) that specializes in breast imaging has been developed, and basic and clinical studies using dbPET are underway [7,8,9]. dbPET can acquire data with higher spatial resolution than whole-body PET, thereby demonstrating higher sensitivity for BC detection [10]. Visual classification of FDG uptake patterns is useful in distinguishing benign from malignant lesions because dbPET can evaluate lesion shape and distribution in more detail than whole-body PET [11]. Several studies have also used the standardized uptake value (SUV), which indicates the degree of FDG uptake, to quantitatively assess the grade of BC and predict chemotherapy response [12,13]. However, dbPET has an issue of high radiation exposure to patients compared with other imaging modalities for BC screening. Repeated exposure may also increase the likelihood of developing malignant tumors over a lifetime [14]. This problem may be solved by reducing the drug dosage; however, poor diagnostic performance due to reduced image quality is a concern [15].

Artificial intelligence (AI) is being increasingly used in medical imaging applications [16,17,18,19]. AI has demonstrated excellent performance in pattern recognition, segmentation, object detection, and image synthesis in BC imaging [20,21,22,23,24]. AI is also expected to achieve high diagnostic accuracy and affect clinical practice in nuclear medicine [25,26,27,28].

Recently, an AI-based image generation model called the generative adversarial network (GAN) [29], which generates realistic virtual images by competing discriminators with generators, has attracted attention. Pix2pix is a type of GAN that transforms one image to another by learning pairs of images [30]. Several studies have revealed that pix2pix-generated synthetic images may be useful in clinical practice; moreover, their clinical applications and use as educational data have been proposed [31,32]. High-quality synthetic images generated from low-quality dbPET images using GAN may overcome the problems related to radiation exposure in dbPET because images acceptable for diagnosis are obtained even with a significantly reduced injection dosage.

This study aimed to determine the feasibility of synthetic dbPET images generated from low-quality images using the pix2pix model by learning pairs of low- and full-count dbPET images for clinical practice by visual and quantitative evaluation.

## 2. Materials and Methods

### 2.1. Participants

This retrospective study was approved by the review board of the Kofu Neurosurgical Hospital (approval date: 12 October 2021), which waived the requirement for informed consent. From April 2015 to August 2020, 1598 dbPET examinations were performed on women at our hospital. This study randomly selected 49 breasts from 45 patients after excluding patients previously treated for BC. The average age of the patients was 57.4 (range, 27–86) years.

### 2.2. Imaging Protocol

**Figure 1:**
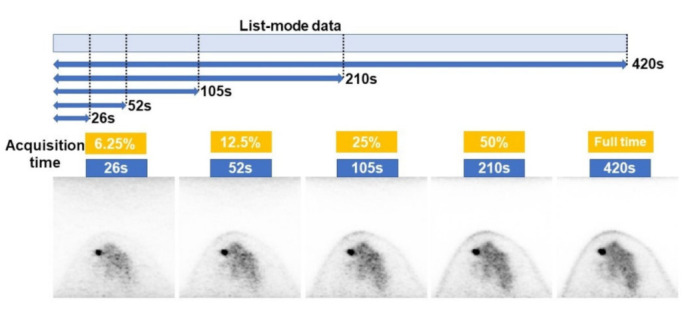
Image reconstruction with divided list-mode data. Images reconstructed from data acquired for 420 s. The list-mode data of the breast were divided into 26 s (6.25%), 52 s (12.5%), 105 s (25%), and 210 s (50%) from the start and reconstructed.

All patients received ^18^F-FDG (3 MBq/kg) after fasting for at least 6 h, and dbPET scanning was performed for 7 min for each breast in the prone position approximately 90 min after ^18^F-FDG injection. The dbPET scanner used herein was Elmammo (Shimadzu, Kyoto, Japan), which consisted of 36 detector modules arranged in three contiguous rings with a diameter of 195 mm, an axial length of 155.5 mm, and interaction depth measurement capability. The settings of these reconstruction parameters were defined in a previous report [33]. A total of 236 transverse dbPET images with 7.8 mm slice thickness were obtained for each breast. dbPET images with short acquisition times were reconstructed from 420 s full-time data divided into 26 s (6.25%), 52 s (12.5%), 105 s (25%), and 210 s (50%) from the start of acquisition (Figure 1).

### 2.3. Final Diagnoses

The diagnoses of dbPET images were classified into two categories, namely, “abnormal” and “normal”. The “abnormal” diagnosis was a breast with localized FDG accumulation that could not be explained via physiologic breast accumulation or noise, regardless of a benign or malignant tumor. A breast radiologist with 12 years of experience reviewed the images concerning the dbPET lexicon [11].

### 2.4. Data Set

This study analyzed dbPET images of 49 breasts from 45 patients, including 32 abnormal and 17 normal diagnoses. Each image was sized to 958 × 940 and windowed between an SUV of 0 and 4 before being converted from Digital Imaging and Communications in Medicine (DICOM) to Portable Network Graphics (PNG). Then, patients were randomly assigned to a training image set (*n* = 16 [normal; *n* = 4 and abnormal; *n* = 12]), validation image set (*n* = 7 [normal; *n* = 2 and abnormal; *n* = 5]), and test image set (*n* = 26 [normal; *n* = 11 and abnormal; *n* = 15]). A total of 3776 images were used for training and 1652 images were used for validation.

### 2.5. Model Description and Training Protocol

The computer (DEEPstation DK-1000; UEI, Tokyo, Japan) that was used in our image synthesis contained a graphic processing unit (GeForce GTX 1080; NVIDIA, Santa Clara, CA, USA), central processing unit (Core i7-8700; Intel, Santa Nlara, CA, USA), and a graphical user interface-based deep learning tool (Deep Analyzer; GHELIA, Tokyo, Japan).

**Figure 2:**
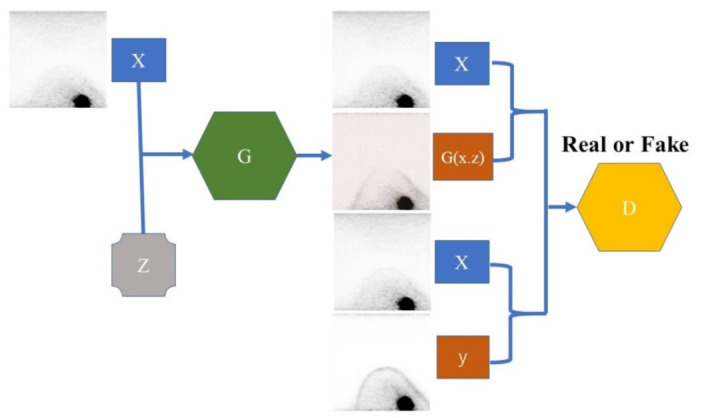
Structure of pix2pix. Generator G generates a synthetic image G (x, z) from an original image x and a noise vector z. Discriminator D classifies whether the “pair of original image x and real image y” and the “pair of original image x and synthetic image G (x, z)” are real. This structure allows Generator G to learn the relationship between image pairs to generate realistic synthetic images from the original images.

The images were constructed with pix2pix, which used conditional GAN for image-to-image translation [30,34]. Pix2pix is an algorithm for image synthesis, which learns a transformation method between two pairs of images and generates a synthetic image corresponding to the input image based on the learning result (Figure 2) [30]. The parameters include an optimizer and discriminator = Adam (lr  =  0.001, beta_1  =  0.9) and a batch size of 64. This model was trained by pairing short- and full-acquisition time dbPET images with 100 epochs. The output of the model is a synthetic image of the full-acquisition time when the input is the short-acquisition time of the test data. The input and output image data were both set at 256 × 256 pixel sizes. The study parameters were based on previous studies [31].

### 2.6. Evaluation of Models

**Figure 3:**
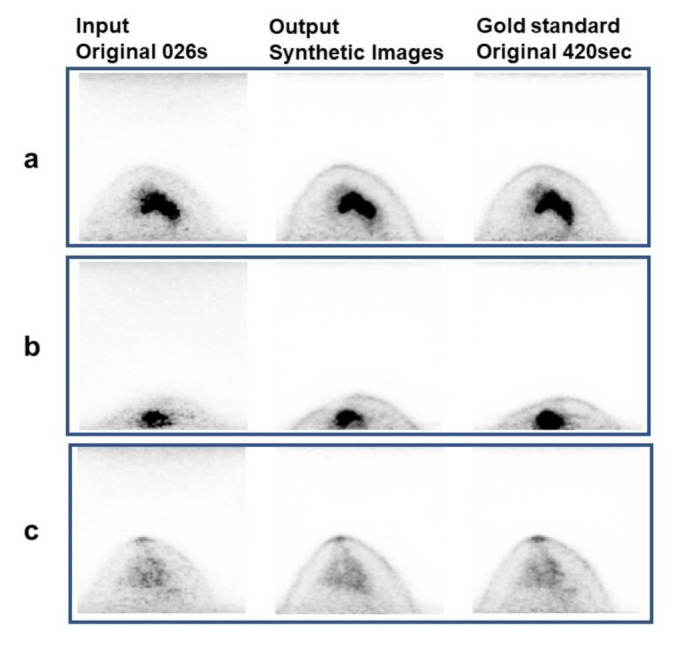
Examples of synthetic images. The left row shows the input (original images), the middle row shows the output (synthetic images), and the right row shows the gold standard (original 420-s acquisition images). (**a**,**b**) Breasts with abnormal accumulation. (**c**) Normal breast.

High-quality dbPET images were synthesized from the test images of 26 breasts with short acquisition times using the developed model (Figure 3). Two representative images from one breast were selected for image evaluation. One image with abnormalities and one image without abnormalities were selected for the breasts showing abnormal ^18^F-FDG uptake.

Two breast radiologists with 8 and 21 years of experience performed visual analysis. Two readers visually evaluated the original low-count time image side-by-side randomly with the synthetic image produced by our model with short-acquisition time by referring to the full-count image, which is the gold standard. They were blinded to the original and synthetic images as well as to the acquisition time. Image quality was rated on a scale of 1 to 5 (1, the left image is much better than the right; 2, the left image is better than the right; 3, the left image and right image are comparable; 4, the right image is better than the left; and 5, the right image is much better than the left). The following parameters were evaluated: (1) smoothness (less noise), (2) anatomical clarity of the breast (such as mammary gland, fat, and nipple), and (3) overall image quality. The visibility of abnormal lesions in the dbPET images was also assessed in 15 cases with abnormal FDG accumulation.

Quantitative analysis was performed by comparing the structural similarity index measure (SSIM) and peak signal-to-noise ratio (PSNR) of the original short acquisition time image and the synthetic image produced by our model to those of the full-acquisition time image.

### 2.7. Statistical Analysis

The scores obtained from the visual assessment were modified by a third party as follows: 1, the original image is much better than the synthetic image; 2, the original image is better than the synthetic image; 3, both images are comparable; 4, the synthetic image is better than the original image; and 5, the synthetic image is much better than the original image. All statistical analyses were conducted using IBM SPSS for Windows (version 24.0; IBM Corp., Armonk, NY, USA). Interobserver agreement was evaluated using weighted kappa. Kappa coefficients were calculated by comparing the five-point scale evaluation scores of the two readers and interpreted as slight (<0.20), fair (0.21–0.40), moderate (0.41–0.60), substantial (0.61–0.80), and almost perfect (0.81–1.0) [35]. The PSNR and SSIM of short-acquisition original images, full-count images, and their synthetic images followed a normal distribution. Thus, the PSNR and SSIM were analyzed with a corresponding *t*-test. A *p*-value of <0.05 was considered statistically significant.

## 3. Results

### 3.1. Visual Analysis Results

**Figure 4:**
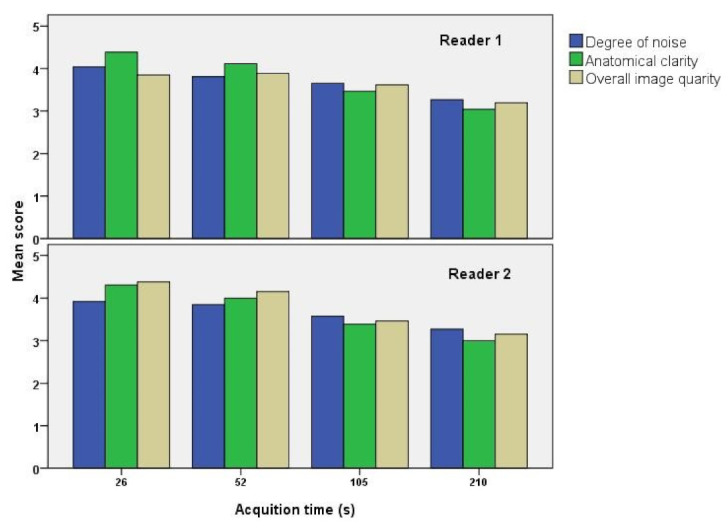
Quantitative analysis of the synthetic and original images. The two readers visually evaluated the degree of noise, anatomical clarity, and overall image quality of the synthetic images. The average score for all factors was >3, suggesting that the synthetic images had better image quality than the original images with shorter acquisition times. In addition, synthetic images generated from images with shorter acquisition times had higher average scores, suggesting that our image synthesis model is more effective for lower quality images with shorter acquisition times.

Table 1 and Figure 4 present the results of the visual analysis of synthetic images performed by two readers in terms of smoothness (low noise), anatomical clarity, and overall image quality. The interobserver agreement between the two readers by weighted kappa was K = 0.356 (fair), K = 0.577 (moderate), and K = 0.359 (fair) for smoothness, anatomical clarity, and overall image quality, respectively. The average scores for all parameters were >3, suggesting that the synthetic images generated by our model had better image quality than the original images with short-acquisition time. Furthermore, the average score was higher for synthetic images derived from images with shorter acquisition times, suggesting that our image synthesis model is more effective in improving lower image quality. Regarding images with longer acquisition times, although the smaller standard deviation of visual scores for synthetic images indicated more stable quality, it suggested that the model could only produce minimal improvement in image quality.

### 3.2. Quantitative Analysis Results

Table 2 presents the quantitative analysis results of the original and synthetic images using SSIM and PSNR as similarity indicators. Both SSIM and PSNR values increased with an increase in acquisition time for both the original and synthetic images. The synthetic image demonstrated significantly higher SSIM and PSNR than the original image for the 26 s acquisition image (*p* < 0.01 and *p* < 0.01, respectively). The synthetic image demonstrated significantly higher PSNR than the original image for the 52 s acquisition image (*p* < 0.01). Conversely, SSIM and RSNR were higher for the original image than those for the synthetic image in the 105 s and 210 s acquisition images, with significant differences in SSIM for the 105 s acquisition image (*p* < 0.01) and in SSIM and RSNR for the 210 s acquisition image (*p* < 0.01 and *p* < 0.01, respectively). These results were consistent with the visual evaluation results, wherein longer acquisition time images had a smaller effect on image quality improvement by the model.

### 3.3. Detection Rate of Abnormal Accumulation

Table 3 shows the comparison results of the detection rates of abnormal accumulation between the original and synthetic images. Almost no difference was observed in the detection rate of abnormal lesions in the original and synthetic images. The detection rate of lesions was higher for the original and synthetic images with a longer acquisition time. Moreover, the acquisition time of 210 s resulted in 100% detection of lesions in both the original and synthetic images.

**Figure 5:**
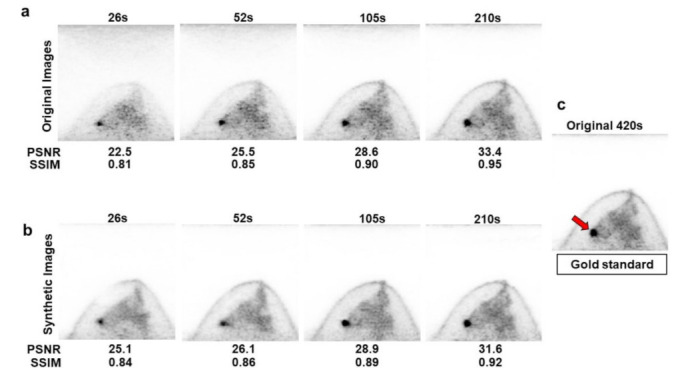
Representative case of abnormal accumulation on dbPET. The original (**a**) and synthetic (**b**) dbPET images for each acquisition time are shown with the values of the peak signal-to-noise ratio (PSNR) and structural similarity (SSIM). Abnormal accumulation in the breast is shown in the original 420 s image ((**c**), arrow). The synthetic images were visually evaluated to have better dbPET image quality than the original images, and the visual image quality improvement was higher for the images with shorter acquisition times (026 s and 052 s). The PSNR and SSIM were also quantitatively improved for dbPET images with shorter acquisition times (026 s and 052 s). The detection of abnormal lesions was almost the same for the original and synthetic images at all acquisition times.

Figure 5 illustrates the representative synthetic images generated by the model used in this study.

## 4. Discussion

Respiratory motion is suppressed because dbPET is performed in the prone position with the breast naturally hanging and extending, thereby allowing high-resolution breast PET images to be captured with almost no blurring. However, structures with FDG accumulation continuously exist from the breast within the field of view (FOV) to the torso outside the FOV, resulting in increased noise at the edge of the FOV in dbPET images. Furthermore, parameters in image reconstruction from raw data acquired with dbPET were determined to optimally balance small lesion detectability with noise in background mammary glands. This increases noise in dbPET images compared with whole-body PET images. These noises are sometimes difficult to distinguish from small lesions and can lead to false-negative or false-positive findings, which affect quantitative PET values such as SUV [7,24]. Increasing the acquisition time and injecting a sufficient FDG dose, which lead to increased patient burden and decreased test efficiency, are necessary to reduce noise.

This study revealed that pix2pix, an image-to-image conversion, can improve the image quality of dbPET with short acquisition times. This is consistent with the following achievement revealed in subsequent studies on medical image processing using pix2pix. Mori et al. developed a model for generating fat-saturated T1-weighted image (T1WI) synthesis from T1WI of magnetic resonance imaging (MRI) using pix2pix. They successfully generated images with homogeneous fat suppression, reduced artifacts, and concluded that pix2pix can generate fat-saturated T1W1 synthesis in breast MRI [31]. Ueda et al. acquired 17,934 image pairs collected from 40 patients to generate cerebral vascular images with few artifacts using pix2pix. Their synthetic images demonstrated high agreement with digital subtraction angiography (DSA) images as the gold standard, with mean PSNR and SSIM of 40.2 dB and 0.97, respectively. All readers assessed the synthetic angiograms generated as more clinically useful than the misregistration DSA original images on visual evaluation. Image transformation and generation using deep learning shows great promise for medical imaging applications and clinical use, as previously mentioned [32]. In addition, studies have reported other image transformation and quality improvement techniques for CT and MRI using deep learning [36,37,38,39,40].

Some studies have attempted to improve the quality of low-dose PET images with deep learning techniques similar to ours. Chen et al. reported that full-dose-like amyloid PET images can be generated from MRI and 100-fold fewer counts of ultralow-dose PET data [41]. Wang et al. developed a model to generate diagnostic 18F-FDG PET images from ultralow-dose 18F-FDG PET input images for patients with pediatric cancer. Their model could generate simulated clinical standard 18F-FDG PET images while maintaining clinically relevant information, such as diagnostic accuracy and quantitative SUV measurements [42]. Recently, it has been expected to reduce exposure dose by improving image quality using deep learning technology.

The present study revealed that all factors (i.e., degree of noise, anatomical clarity, and overall image quality) scored > 3 by both experts in the visual evaluation, proving the visual image quality improvement effect of dbPET with synthetic images. This improvement effect was higher for low-quality images (026 s and 052 s) with shorter acquisition times. SSIM and PSNR increased for images with short acquisition times (026 s and 052 s) in the quantitative evaluation, as in the visual evaluation. Conversely, SSIM and PSNR decreased with longer acquisition times (105 s and 210 s). Notably, these images (105 s and 210 s) exhibited a quality improvement visually but not quantitatively. This result suggests that the model is more effective when the image quality is poorer. Previous studies have revealed the usefulness of pix2pix image transformation in cases of poor image quality, such as those with motion artifacts [32,36], which is consistent with our results. Shortened acquisition times are expected to have a synergistic effect by increasing patient compliance and reducing movement artifacts.

Images with shorter acquisition time, which were more effective in improving image quality by the present model, are images with fewer RI counts. Thus, even images with fewer RI counts caused by reduced FDG dose can be improved. A previous study on actual low-dose dbPET revealed that a clinically acceptable image quality is obtained at approximately 25–50% of the dose for conventional whole-body FDG PET [15]. In this study, images were sampled from the start time of collection; thus, the earlier the start time of acquisition, the higher the RI counts, and the later the start time of acquisition, the lower the RI counts, even within the same minute. In other words, acquisition times are not strictly linearly related to RI counts. To obtain the same RI counts, one might theoretically need to calculate and sample the same number of seconds. However, this is a very complicated method, and in practice, it is difficult to do in each case. Therefore, we chose this simple method used in a previous study [15]. Accordingly, we can approximately assume that (A% acquisition times) × (B% dose) image” = “(B% acquisition times) × (A% dose) image. In this study, the data, for example, with a 26 s acquisition time, can be regarded as an image with an FDG dose of approximately 6.25% of the clinical dose. Specifically, our deep learning model reveals that even low-dose images at 6.25% of the conventional clinical dose are obtained with image quality comparable with that of full-dose images. The results suggest a significantly reduced patient exposure to dbPET.

The detection rate of abnormal lesions was almost the same for both the original and generated images, which may reflect that the synthetic images were generated by distinguishing between noise and lesions and reducing the noise only. In this study, the model was trained on a relatively small number of cases, but a more sophisticated model could be built by training on a larger number of data sets, which may improve image quality and lesion detection rates. Clinical applications of this synthetic image technology could shorten acquisition times and reduce PET doses, making dbPET more practical in the future. However, the interobserver agreement between the two readers in the visual analysis was K = 0.356–0.577 (fair to moderate). To allow the clinical application of dbPET synthetic images, it may be necessary to refine the image evaluation criteria, such as using common finding terms [11] to describe the characteristics of dbPET synthetic images.

This study had some limitations. First, this was a retrospective study conducted at a single institution with relatively small cohort size. Second, this study was conducted using images converted from DICOM and PNG, and the image data were set at an input size of 256 × 256 pixels and an output size of 256 × 256 pixels. This image processing may have resulted in information loss and influenced its performance. SUV is useful for predicting the prognosis and determining the efficacy of treatment for BC. Although SUVs were not considered in this study, the influence of SUV on synthetic images should be examined in the future.

In conclusion, our image-to-image translation model can be used to generate improved quality short-time acquisition dbPET images without affecting the lesion detection rate. Both visual and quantitative evaluations revealed that the dbPET image quality improvement was more effective for lower quality images with shorter acquisition times.

## Figures and Tables

**Table 1:** Visual analysis of the synthetic and original low-count images.

	Degree of Noise (Mean ± SD)	Anatomical Clarity (Mean ± SD)	Overall Image Quality (Mean ± SD)
Reader 1			
26 s	4.04 ± 0.71	4.38 ± 0.74	3.85 ± 1.23
52 s	3.81 ± 0.73	4.12 ± 0.85	3.88 ± 0.97
105 s	3.65 ± 0.55	3.46 ± 0.69	3.62 ± 0.68
210 s	3.27 ± 0.44	3.04 ± 0.34	3.19 ± 0.39
Reader 2			
26 s	3.90 ± 0.62	4.31 ± 0.72	4.38 ± 0.74
52 s	3.85 ± 0.53	4.00 ± 0.68	4.15 ± 0.66
105 s	3.58 ± 0.49	3.38 ± 0.49	3.46 ± 0.50
210 s	3.27 ± 0.44	3.00 ± 0.00	3.15 ± 0.36

SD, standard deviation. Scores: 1, the original image is much better; 2, the original image is better; 3, equal; 4, the synthetic image is better; and 5, the synthetic image is much better.

**Table 2:** Quantitative analysis of the synthetic and original images.

Acquisition Time	Image Type	SSIM (Mean ± SD)	*p*-Value	RSNR (mean ± SD)	*p*-Value
026 s	Original	0.83 ± 0.08	<0.01	23.9 ± 2.4	<0.01
	Synthetic	0.85 ± 0.07		27.2 ± 3.6	
52 s	Original	0.88 ± 0.06	0.16	26.8 ± 2.8	<0.01
	Synthetic	0.87 ± 0.07		28.3 ± 4.0	
105 s	Original	0.92 ± 0.04	<0.01	30.2 ± 3.2	0.62
	Synthetic	0.90 ± 0.05		30.0 ± 4.3	
210 s	Original	0.96 ± 0.02	<0.01	35.0 ± 2.3	<0.01
	Synthetic	0.93 ± 0.03		32.4 ± 3.9	

PSNR, peak signal-to-noise ratio; SD, standard deviation; SSIM, structural similarity index measure.

**Table 3:** Comparison of the detection rates of abnormal accumulation between the original and synthetic images.

	Original Images	Synthetic Images
Reader 1		
26 s	*n* = 10/15 (66.7%)	*n* = 9/15 (60.0%)
52 s	*n* = 14/15 (93.3%)	*n* = 13/15 (86.7%)
105 s	*n* = 14/15 (93.3%)	*n* = 13/15 (86.7%)
210 s	*n* = 15/15 (100%)	*n* = 15/15 (100%)
Total	*n* = 53/60 (88.3%)	*n* = 50/60 (83.3%)
Reader 2		
26 s	*n* = 7/15 (46.7%)	*n* = 9/15 (60.0%)
52 s	*n* = 12/15 (80.0%)	*n* = 12/15 (80.8%)
105 s	*n* = 14/15 (93.3%)	*n* = 14/15 (93.3%)
210 s	*n* = 15/15 (100%)	*n* = 15/15 (100%)
Total	*n* = 48/60 (80.0%)	*n* = 50 (83.3%)
Reader 1 and 2 (mean)		
26 s	*n* = 8.5/15 (56.6%)	*n* = 9/15 (60.0%)
52 s	*n* = 13/15 (86.7%)	*n* = 12.5/15 (83.3%)
105 s	*n* = 14/15 (93.3%)	*n* = 13.5/15 (90%)
210 s	*n* = 15/15 (100%)	*n* = 15/15 (100%)
Total	*n* = 50.5/60 (84.2%)	*n* = 50/60 (83.3%)

The number of cases detected from 15 cases with abnormal accumulation and the detection rate (%) are shown.

## Data Availability

Not applicable.
